# Supplementary material for: Impact of Local Stiffness on Entropy Driven Microscopic Dynamics of Polythiophene
Source: Sci Rep. 2020 Jun 19;10:9966. doi: 10.1038/s41598-020-66354-6 (PMC7305133; doi:10.1038/s41598-020-66354-6)
Supplement: Supplementary file 1 — Supplementary Information for: Impact of Local Stiffness on Entropy Driven Microscopic Dynamics of Polythiophene. [file 41598_2020_66354_MOESM1_ESM.pdf]

Supplementary Information for:

Impact of Local Stiffness on Entropy Driven Microscopic Dynamics of Polythiophene

Sudipta Gupta,[1,*] Sourav Chatterjee,[1] Piotr Zolnierczuk,[2] Evgueni E. Nesterov,[1,3*] and Gerald J. Schneider[1,4*]

[1]*Department of Chemistry, Louisiana State University, Baton Rouge, LA 70803, USA*

[2]*Jülich Centre for Neutron science (JCNS) outstation at SNS, POB 2008, 1 Bethel Valley Road, TN 37831, Oak Ridge, USA*

[3]*Department of Chemistry and Biochemistry, Northern Illinois University, DeKalb, IL 60115, USA*

[4]*Department of Physics and Astronomy, Louisiana State University, Baton Rouge, LA 70803, USA*

Viscosity

We obtained, the solvent viscosity, η_s, for deuterated 1,2-dichlorobenzene (DCB-D_4) by linear interpolation from the tabulated viscosity.[1] It is illustrated in figure SM1.

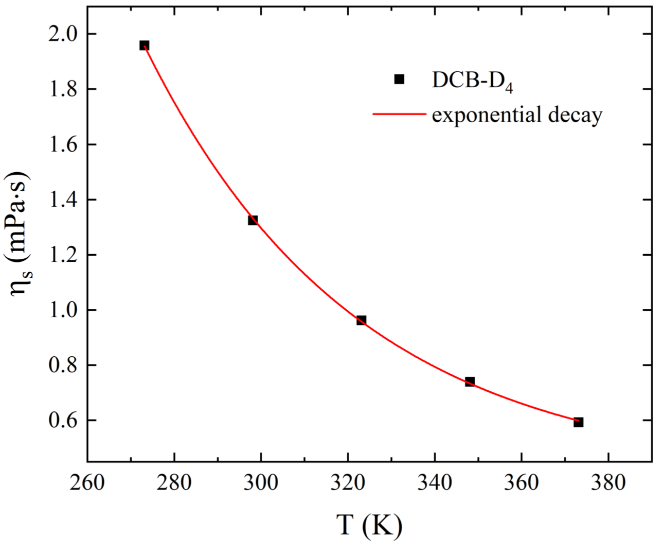

Figure SM1. Viscosity, η_s, of DCB-D_4 solvent as a function of temperature (T). Data obtained from tabulated data in the literature. [1]

UV/vis Absorption Spectroscopy

Variable-temperature UV/vis absorption spectra were recorded on an Agilent Cary 5000 UV-Vis-NIR spectrometer equipped with Peltier temperature-controlled sample holders. Figures SM2 (a-b) illustrate change in absorption spectra of the two P3HT samples in DCB at different temperatures.

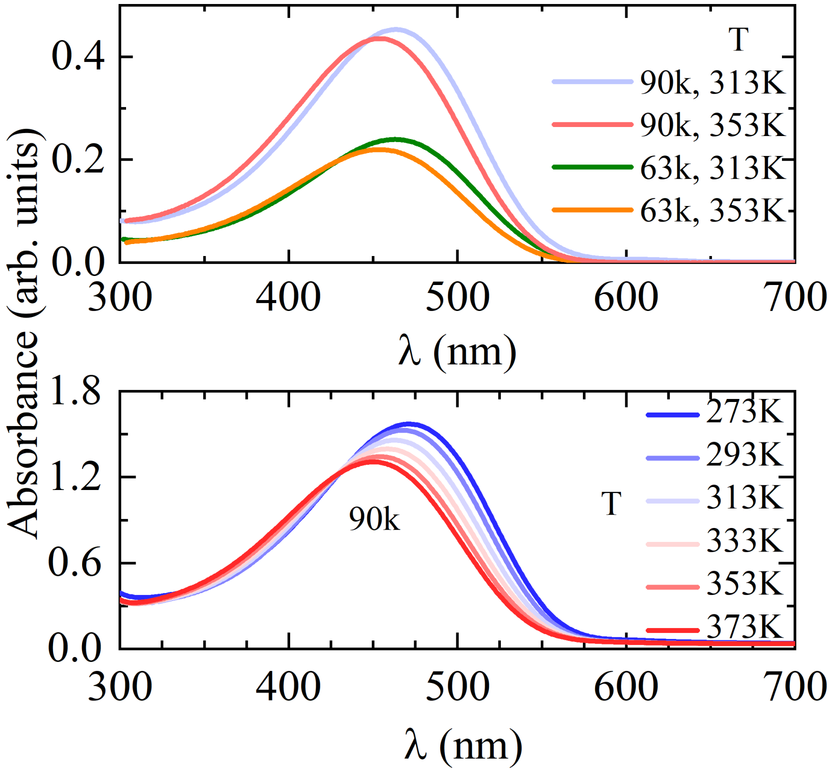

Figure SM2. (a) Steady-state absorption spectra of two samples of P3HT in DCB (concentration 0.0015 wt.%) obtained at the two different temperatures used in this study. (b) Temperature dependence of the absorption spectra for 90 kg/mol P3HT sample.

Chain Conformation

Small angle neutron scattering (SANS) is used to determine the chain conformation as shown in Figure SM3.

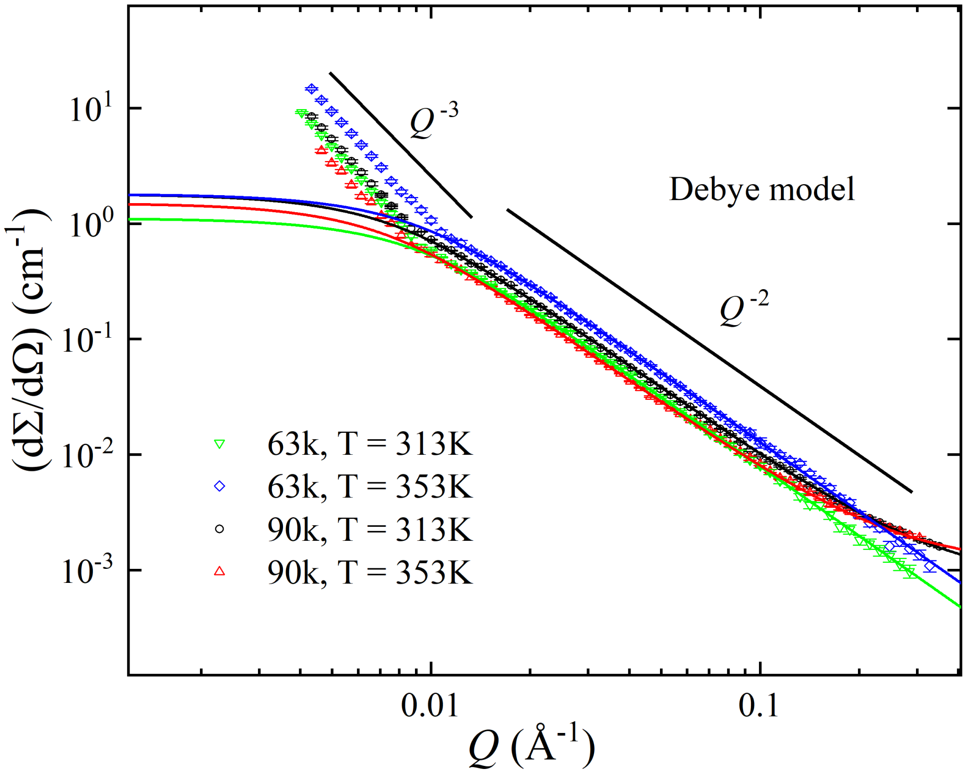

Figure SM3. SANS scattering intensity as a function of momentum transfer, Q, for two different molecular weights and temperatures as indicated in the legends. The power-law dependence is illustrated. The solid lines represent the Debye model as explained in the manuscript.

Chain Dynamics Analysis

From NSE spectroscopy the chain dynamics cannot be only explained from the calculated Zimm-diffusion, D_Z, Zimm-time, τ_Z, for the chain end-to-end distance, R_{ee}, as obtained independently from SANS. Figure SM4 represents the data. The corresponding simulated data for different modes like p = 1, 5, and 10, respectively, are presented in Figure SM5. It illustrates that for different Q's the simulated data can give a better description of the experimental data only by adjusting p. This systematic variation yields the best description for p_{min} as presented in the manuscript.

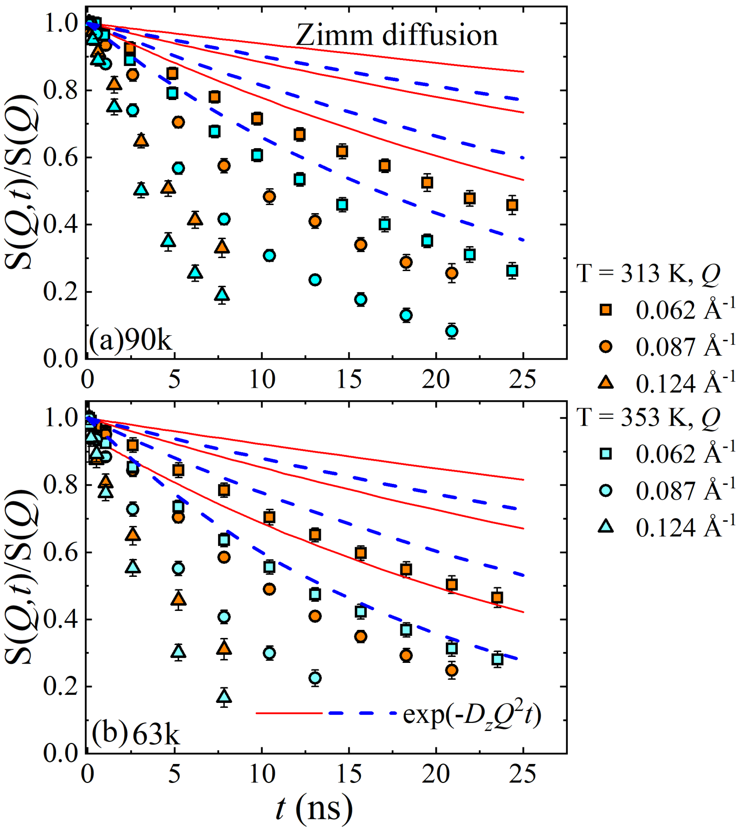

Figure SM4. Normalized dynamic structure factor (S(Q,t)/S(Q)) as a function of Fourier times (t) for different M_n = 90 kg/mol (a) and 63 kg/mol (b) and T = 313K (red) and 353K (cyan), respectively. The different momentum transfer, Q, is given by, □ = 0.062 $Å^{-1}$, ○ = 0.087 $Å^{-1}$ and △ = 0.124 $Å^{-1}$. The solid lines in (a) and (b) represents the center of mass Zimm diffusion, $exp(-Q^2 D_Z t)$, *from Eq. (1) in the manuscript.*

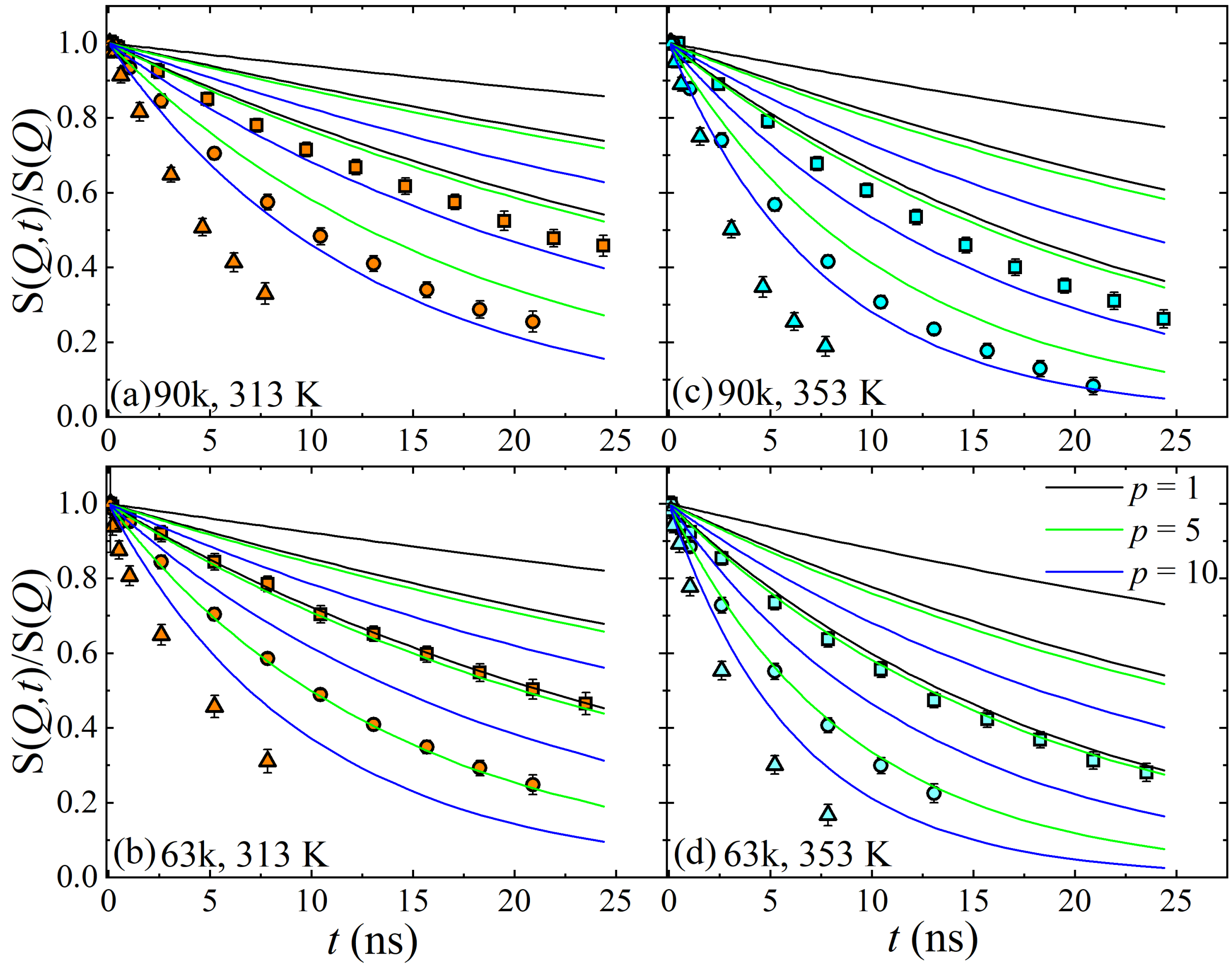

Figure SM5. Normalized dynamic structure factor (S(Q,t)/S(Q)) as a function of Fourier times (t) for different M_n and temperatures. (a) 90 kg/mol, 313 K; (b) 63 kg/mol, 313 K; (c) 90 kg/mol, 353K; (d) 63 kg/mol, 353 K. The different momentum transfer, Q, is given by, □ = 0.062 Å^{-1}, ○ = 0.087 Å^{-1} and △ = 0.124 Å^{-1}. The solid lines represent the simulated data for different modes, p = 1 (black), 5 (green) and 10 (blue), for Q = 0.062 Å^{-1}, 0.087 Å^{-1}, and 0.124 Å^{-1}, respectively, from top to bottom.

References

1 Haynes, W. M. *CRC Handbook of Chemistry and Physics*. 93 edn, (CRC, 2012).
